# Neuromodulatory roles of *PIPER GUINEENSE* and honey against Lead-Induced neurotoxicity in social interactive behaviors and motor activities in rat models

**DOI:** 10.3934/Neuroscience.2022026

**Published:** 2022-11-15

**Authors:** UCHEWA O. Obinna, EMECHETA S. Shallom, EGWU A. Ogugua, EDE C. Joy, IBEGBU O. Augustine

**Affiliations:** Department of Anatomy, Faculty of Basic Medical Sciences, College of Medicine, Alex Ekwueme Federal University Ndufu-Alike, Ikwo (AE-FUNAI), Ebonyi State, PMB 1010, Nigeria

**Keywords:** lead aacetate, behavior, motor activities, neuromodulator, neurotoxicity, social interaction

## Abstract

**Background:**

*Piper guineense* and honey contain antioxidative, anti-inflammatory, and antimicrobial properties that can help restore neuronal and other cell damage. To investigate the neuromodulatory roles of *p. guineense* and honey against lead toxicity on the hippocampus and cerebellum, impairing social behaviors and motor activities.

**Methodology:**

Thirty Wistar rats were separated into six groups of five rats each, marked with dye. Group A served as control; B was untreated lead; C was a medium dose of the extract (50 mg/kg) and honey (1000 mg/kg); D was a high dose of the extract (80 mg/kg) and honey (1500 mg/kg); E received extract (80 mg/kg), and F received honey (1500 mg/kg). All groups received 110 mg/kg of lead orally, except the control. Social interaction, antidepressant effects, and motor activities were studied using a sociability chamber (SC), Forced Swim Test (FST), and String methods. A blood sample was used to evaluate glutathione peroxidase (GPx) and glutathione oxide transaminase (GOT), while the lipid level was estimated using cerebellar homogenate. Neuronal damage, vacuolation, necrosis, cell degeneration, and alterations in both hippocampus and cerebellum marked untreated group, with decreased GPx and GOT activities followed by impaired motor activities, social behavior, memory, and motivation. Using SCT, group B spent significantly lesser time (47.60 ± 47.60) with stranger 1 compared to A (138.20 ± 34.05), while group C spent considerably more time with stranger 1 (86.80 ± 30.32) than group B at P ≥ 0.05. The treatment increased the enzyme level and restored histoarchitecture (Figures 1–12), improving motor activities, social behavior, memory, motivation, and social affiliation (Tables 3, 4, 2, and 6). The extract and honey may be helpful as neuromodulators in lead toxicity in a dose-dependent manner.

## 1. Introduction

Several neuropsychiatric diseases, including autism spectrum disorders, schizophrenia, bipolar disorders, and obsessive-compulsive disorders, have been linked to social and behavioral disorders, memory disorders, loss of motor activities, and depression [1]. Individual social recognition is critical in stabilizing and improving structural relationships, which are essential in any working society [2]. In lower animals (rodents), social recognition has been identified as a vital means of maintaining social hierarchy and choosing mates [3]. Researchers have discovered that engaging in integrative research with animals using suitable testing techniques for social behavior can proffer the long-awaited solution to social psychopathologies that are ravaging the universe [4]. Memory is an integral part of our social lives that we cannot do without because it allows us to keep records of past events, maintain relationships, create awareness, and improve our interactions with others. Memory loss has the potential to impair our social behaviors and interactions with one another [5]. Some individuals are known to learn faster and usually retain information at the onset. Still, as time goes by, it rapidly disappears at a phenomenal rate, a condition known as accelerated long-term forgetting, which can occur in both the aged and young [6]. Depressed individuals already have impaired social behavior, which is highly likely to impede their interaction with their environment, thereby reducing their social relationships. This condition is capable of distracting and causing them to lose track of records of events within their environment [7]. According to Scola et al. [8], the level of motor activity can be significantly lower in a stranger condition than in the presence of a familiar counterpart.

The hippocampus is essential not only for cognition and learning but also for spatial navigation and emotional behavior [9]. Its critical role in reconstructing relational memory underlies flexible cognition, and social behavior can never be undermined. Any alteration produces high deficits and risks in real-world situations [10]. The hippocampus is the easiest, earliest, and most severely altered structure in neuropsychiatric disorders caused by intoxications such as lead, and that alteration threatens learning and memory [11]. The little brain (cerebellum) is a vital component of the human brain that plays a significant role in muscle coordination, maintenance of body equilibrium, motor functions, and motor learning [12]. Alteration in this part of the brain can lower muscle tone, cause body imbalance, and impede coordination in general.

Lead is one of the most toxic environmental substances; it acts on the central nervous system, causing severe neurobehavioral and psychological alterations [13]. Lead causes neurological defects, developmental delays, learning disabilities, and behavioral abnormalities and is identified as a neuro-developmental toxin [14]. Its neurotoxicity has been shown to include social and behavioral defects, impaired performance, and deficits in motor skills, which are highly likely to persist even after the concentration returns to normal [15]. The knowledge of the neuro-deficits caused by lead is vital to broadening our horizons, and many researchers have focused on it for a long time. This research focuses on discovering how our daily household condiments, like *Piper guineense* and honey, can combine to help restore any impaired social behavior and motor activities caused by neurotoxins. P. guineense and honey were chosen to treat this common toxicity because both are common and essential household ingredients in this part of the world. They are also very affordable, easily assessable, and consume little time to prepare for consumption.

## 2. Methodology

### 2.1. Ethical Clearance

The procedure for taking care of research animals was approved by the research ethic committee of Alex Ekwueme Federal University Ndufu Alike Ikwo (AE-FUNAI) is strictly adhered to. Accordingly, Alex Ekwueme Federal University Ndufu-Alike, Ebonyi State, Nigeria (AE-FUNAI) Animal Use and Research Ethical Committee, gave the ethical certificate with Reference Number AE-FUNAI-2022/00224. As a result, the animal's suffering was highly minimized as all international protocols were obeyed.

### 2.2. Lead Acetate and Honey

Sigma Aldrich, USA, provided lead acetate with a molecular weight of 379.34, batch number CSA200112, product number L005112, and pack size 500 g.with, the brand name-Laser pure blossom honey, and a net weight of 1.0 kg, purchased from a grocery store.

### 2.3. Collection, Identification, and Extraction

*Piper Guineense* leaves were obtained from a herbarium at the University of Nigeria, Nsukka, Enugu State, and identified by the same school with a voucher number of UNN 12. The leaves were dried at room temperature for two weeks to ensure proper water removal. The aqueous extract was obtained following the Soxhlet method described by Zhang et al. [17]. The leaves were crushed into a fine powder using an attrition mill. Extraction was done using the soxhlet apparatus. The 250 ml of the extraction solvent (water) was added to a round-bottom flask and attached to a soxhlet extractor and condenser on a heat source. The fine powder was placed inside the soxhlet thimble made of solid filter paper. The heat was applied to the solvent below the flask for evaporation, passing through the condenser, where it condensed and flowed down to the extraction chamber to begin extraction. The condensate then dripped into the reservoir containing the thimble. The solvent and the extract flowed back to the flask, and the cycle began when the solvent level in the extraction chamber reached the top of the siphon and continued until the components were entirely extracted.

### 2.4. Protocol for Experimentation

The thirty male Wistar rats weighing 150 and 200 g used in this research were obtained from the animal house of Alex Ekwueme Federal University Ndufu Alike, Ikwo, and housed in cages under standardized conditions and allowed free access to feed and water. After a week of acclimatization, the rats were randomly divided into six groups of five animals per group and marked for identification using dye. Group A served as control. B served as the untreated lead group. C was treated with extract and honey (medium dose). D received treatment with extract and honey to serve as a high dose. Group E received lead and was treated with extract alone, while group F received lead acetate and was treated with only honey. Both the induction and treatment were given orally using an oral gavage once daily (mornings) for seven days and 21 days, respectively, based on the individual weights of the rats. The animals were observed strictly after each administration for changes and sacrificed 24 hours after the last administration. Previous studies have established the LD50 of *Piper guineense* to be 100 mg/kg [17]. Based on the reported oral LD50 of lead acetate, which was 550 mg/kg body weight for Wistar rats [18]. In this present study, we administered 20% of lead LD50 to the rats while 40 and 80% of *Piper guineense* were administered as medium and high doses, respectively.

### 2.5. Assessment of Social Behavior

The Sociability Chamber Test (SCT) was used to assess the rat's social and behavioral ability, spatial learning, and memory. It was conducted twice during the study: after the induction of lead acetate and after treatment. There were three sessions within the rectangular chamber, with openings connecting the chambers and two small cages in each of the two lateral chambers [19]. This test was based on the principle of the free will of choice by the subject to explore the compartments during experimental sessions [20]. During the habituation or familiarization session (H/FS), the rat was allowed in an empty chamber to explore it for 10 minutes and familiarize itself with the environment. Next, in the Social Affiliation Session (SAS), an intruder was introduced in a cage in one of the lateral chambers while the other lateral chamber was empty. Finally, during the Social Novelty Session (SNS), the rat encountered a stranger in the previously empty chamber and the familiar rat. The parameters measured include time spent in close contact with a stranger rat, time spent with the intruder, time spent in each chamber, and the number of entries into each chamber.

### 2.6. Assessment of Motor Activities

The experimental animals' grip strength and limb impairment are used to assess motor activity and the number of lesions on the cerebellum. The apparatus is made of 2 mm by 35 cm wire (diameter and length) hung between two poles at 50 cm high over a 6inch cushion support. The rat was allowed to hold on with its forepaws until it lost grip or for a maximum of 180 seconds. The length of time the rat could hold the wire till it fell was recorded, with a cut-off time of 180 seconds. According to the Tariq et al. [21] technique, latency to grip loss is considered an indirect measure of grip strength. To assess the limb impairment, the experimental rats were scored 3 for gripping the wire with both hind paws, 2 for gripping the wire with 1 hind paw, and 1 for not grasping the wire with either hind paw.

### 2.7. Assessment of antidepressant effects

According to Porsolt et al. [22], the Forced Swim Test (FST) was developed to measure the antidepressant effects of compounds in rodents. The rats were made to swim in a 21 by 50 cm (diameter and height) transparent glass vessel containing water to a depth of 26 cm at room temperature (272 °C). The rat was allowed to swim for 300 seconds before it was removed and the water cleaned. The data collected includes the total duration of immobility, duration of struggling, and swimming duration. Immobility is measured when the rats remain floating in the water, except for small movements necessary to keep their heads above the water. The water was changed after each animal to avoid the influence of water temperature and substances left from the previous session. This test was performed twice during the experimental period, such as after the induction of lead acetate and one day after treatment.

### 2.8. Estimation of Brain Biomarkers

Blood samples were collected from the apex of the heart and centrifuged for 1000 RMP (revolutions per minute). The serum was collected to assess the following biochemical parameters: Glutathione peroxidase (GPX) activity and Glutamate Oxaloacetate Transaminase (GOT) activity.

### 2.9. Estimation of the Lipid Profile

After sacrificing, the cerebellum was removed and homogenized. At the same time, the homogenate was dissolved in normal saline to determine the brain concentration of triglycerides, total cholesterol, high-density lipoprotein, and low-density lipoprotein. The concentration of triglycerides (TG) was determined using the GPO-PAP method, according to Cole et al. [23]. In contrast, total cholesterol (TC), high-density lipoprotein (HDL), and low-density lipoprotein (LDL) were determined using the CHOD-PAP technique [23].

### 2.10. Animal Sacrifice

The cervical dislocation method sacrificed the rats 24 hours after the last treatment. The blood samples collected using hematocrit tubes from the angle of the eyes were used for biochemical analysis of brain markers. The lipid profile was measured using a homogenate from the cerebellum of rats. The skull was removed, and the brain was fixed in Bouin's fluid for 48 hours, and after that, the hippocampus was harvested and refixed in 10% formalin for histological studies.

### 2.11. Statistical Analysis

The results were analyzed using the Statistical Package for Social Sciences (SPSS) and expressed as mean Standard Error Mean. A significant difference between the means of groups was determined using a one-way analysis of variance (ANOVA). The significance of the data was established at P ≥ 0.05.

## 3. Results

### 3.1. The Sociability Chamber Test

The table below shows the results of the SCT performed on the rats after the induction of lead acetate. The time group B spent (47.6047.60) with strangers significantly decreased compared to A (138.20 ± 34.05). Furthermore, group C spent more time with stranger 1 (86.80 ± 30.32) than group B at P ≥ 0.05. When stranger 2 was introduced, group B spent more time with it than stranger 1 (see Table 1).

**Table 1.** The effect of exposure to lead toxicity on the Social behavior of Wistar rats.

Groups	TSEC(s)	TSS1(s)	TSS2 (s)
A	156.60±38.93	138.20±34.05	70.60±32.53
B	147.80±55.67*r	47.60±47.60*r	120.20±50.49*
C	175.00±45.72a	86.80±30.32a	86.20±57.94**
D	156.07±52.30a	48.40±30.52	73.40±47.81**
E	67.40±44.57**	158.00±42.59a	64.00±36.69**
F	45.50±36.20**	57.20±48.02a	105.00±59.76**

*Significant increase compared to A at P ≤ 0.05; ** Significant decrease compared to B at P ≤ 0.05; *r Significant decrease compared to A at P ≤ 0.05; a Significant increase compared to B at P ≤ 0.05, **TSS2**-time spent with stranger 2, **TSS2**-time spent with stranger 1 and **TSEC**-time spent in empty cage.

The result showed that group B significantly decreased the time it spent (47.60 ± 47.60) with the stranger 1 rat compared to the control group (131.20 ± 53.31). The time spent with stranger 1 was increased in group C, while group D did not enter the familiar chamber and so did not spend time with stranger 1, but group F spent significantly decreased time (59.00 ± 59.00) with stranger 1. The time spent with stranger 2 was significantly increased in group B (120.20 ± 50.49) compared to group A (58.60 ± 45.15), while reduced time was spent with groups C, D, and E compared to group B (Table 2) at P ≤ 0.05.

**Table 2.** Effect of exposure to lead toxicity and treatment with *Piper guineense* extract.

Groups	TSEC(s)	TSS1(s)	TSS2(s)
A	61.00±44.83	131.20±53.31	58.60±45.15
B	147.80±55.67*	47.60±47.60*y	120.20±50.49*
C	97.60±50.83*r	114.20±44.02**	76.60±42.90*r
D	211.20±28.80**	0.00±0.00*r	91.40±55.98*r
E	57.80±45.64*r	45.20±45.20r	89.20±54.89*r
F	123.25±66.90*r	59.00±59.00**	116.75±66.35*r

* Significant increase compared to A at P ≤ 0.05; ** Significant increase compared to B at P ≤ 0.05; *r Significant decrease compared to B at P ≤ 0.05; *y Significant decrease compared to A at P≤0.05. Note: **TSS2**-time spent with stranger 2, **TSS2**-time spent with stranger 1, **TSEC**-time spent in empty cage.

### 3.2. Limb Impairment

The results showed that animals in group B had a significant reduction in the time spent holding the string after administration of Lead acetate compared to the training time in group A. Groups E and F showed a substantial decrease in the time spent holding the rope at P ≤ 0.05 after they were administered with lead acetate at the dosage of 110 mg/kg compared to group B during the training period. In addition, there was a significant increase in the time the animals spent holding the string at P ≤ 0.05 in group B after treatment with piper guineense leave extract combined with honey when compared to group A. Groups C and D animals showed a significant increase after treatment compared to Group B after the administration (Table 3).

**Table 3.** Effects of *Piper guineense* on limb impairment in rats induced with lead toxicity.

Groups	Training (s)	After administration (s)	After Treatment (s)
**A**	2.30±0.05	1.85±0.21*	2.57±0.10**
**B**	2.10±0.17	1.80±0.08*	2.29±0.14**
**C**	2.20±0.10	1.70±0.10*	2.67±0.07**
**D**	2.03±0.10	1.90±0.06*	2.50±0.13**
**E**	2.27±0.10	1.75±0.15*	2.50±0.86**
**F**	2.43±0.62	1.90±0.17*	2.47±0.15**

*Reduction compared to training period @ P ≥ 0.05; ** Significant increase compared to after induction @ P ≥ 0.05

### 3.3. Grip Strength

The results showed that groups E and F significantly increment in the grip strength after treatment compared to group B after the administration (Table 4). After treatment, group B showed an increase in grip strength compared to group A after the administration. However, after the administration, group B showed a significant increase in grip strength compared to group A at P ≤ 0.05 (Table 4).

**Table 4.** The effect of *Piper guineense* on grip strength of rats administered with lead acetate.

Groups	Training (s)	After induction (s)	After Treatment (s)
**A**	32.87±5.79	44.15±20.29*	32.87±5.79**
**B**	42.33±7.23	38.35±6.73**	37.63±5.02
**C**	23.33±4.20	22.30±6.11	23.33±4.20
**D**	31.37±7.91	15.37±3.14**	31.33±6.59***
**E**	41.03±6.75	32.60±11.55**	39.34±5.80***
**F**	34.27±4.81	19.30±6.98**	37.60±6.30***

*Reduction compared to training period; ** Increment compared to after induction

### 3.4. Forced Swim Test

Animals in group B showed a significant increment in the immobile time compared to group A after administration with lead acetate (P < 0.05). The number of times the animals in group C spent climbing increased significantly compared to the number of times group B animals spent immobile (p < 0.05). There was an increase in the climbing ability of group D animals compared to the number of times group B spent as immobile (P ≤ 0.05). Group B significantly reduced swimming time compared to group A (P ≤ 0.05). Animals in group F showed an increase in the time they spent swimming compared to group B (P ≤ 0.05), as shown in Table 5.

**Table 5.** The effects of lead acetate toxicity on the locomotive abilities of adult Wistar rats using the forced swimming test.

Groups	Immobile time (s)	Struggling time (s)	Swimming time (s)
**A**	96.60±11.84	191.40±17.56	25.00±15.00
**B**	138.20±23.29*	185.80±10.20r*	5.67±2.03r*
**C**	131.20±22.54**	152.40±1252**	15.37±3.50*
**D**	110.40±17.94**	188.40±16.68c	9.33±5.37*
**E**	140.80±12.32*r	179.80±29.19**	15.00±2.65*
**F**	132.60±19.09**	184.00±11.77	7.50±2.50c

* Significant increase compared to A at P ≤ 0.05; ** Significant increase compared to B at P ≤ 0.0; *r Increase compared to B at P ≤ 0.05; r* significant decrease compared to A at P ≤ 0.05

### 3.5. Forced Swim Test after Treatment

There was a significant increase in the number of times animals in group B struggled after the administration compared to group A (P ≤ 0.05). Groups C, D, E, and F showed a significant increase in mobility, struggling time, and reduction in swimming time compared to group B (P ≤ 0.05). In contrast, group D significantly increased compared to group B in swimming time (P ≤ 0.05). Group B significantly increased swimming time compared to group A (Table 6).

**Table 6.** The effects of *Piper guineense* and honey treatment on the locomotive activities of experimental Wistar rats using forced swim test.

Groups	Immobile time (s)	Struggling time (s)	Swimming time (s)
**A**	91.40±11.11	199.60±11.56	2.33±0.89
**B**	102.80±23.12*	211.60±14.93*	6.00±4.00*
**C**	112.40±9.18**	211.40±7.78	3.67±1.20r*
**D**	137.00±17.58**	168.20±10.31**	7.00±3.00c
**E**	170.00±15.79**	169.20±22.70**	3.67±1.45r*
**F**	163.20±18.44**	146.60±19.30**	3.00±1.16r*

* Significant increase compared to A at P ≤ 0.05; ** Significant increase compared to B at P ≤ 0.05; C Increase compared to B at P ≤ 0.05; r* significant decrease compared to B at P ≤ 0.05.

### 3.6. Assay of Brain Markers

The result from the present research showed that the GPx was decreased in the untreated group B (0.82 ± 0.33) compared to the control (1.37 ± 0.25) (P ≤ 0.05). Groups C, D, E, and F showed that the activity level of GPx increased compared to the untreated group B. On the other hand, the activities of GOT were reduced in group B (8.33 ± 0.28) compared to the control group at P ≤ 0.05. The result showed that groups C and E were reduced but were later elevated in groups D and F compared to group B, as seen in Table 7 below.

**Table 7.** The assay of the biomarkers of GPX and GOT activities in the experimental rats.

GROUP	A	B	C	D	E	F
GPx (U/ml)	1.37 ± 0.25	0.82 ± 0.33	1.08 ± 0.27	1.30 ± 0.32	1.36 ± 0.15	1.15 ± 0.17
GOT(U/ml)	8.35 ± 0.15	8.33 ± 0.28*	8.18 ± 0.04**	8.39 ± 0.04*r	8.17 ± 0.15**	8.69 ± 0.06*r

*Significant decrease compared to A at P ≤ 0.01; **Significant decrease compared to B at P ≤ 0.01; *rSignificant increase compared to B at P ≤ 0.01

### 3.7. Lipid Profile

The result showed a reduction in the Total Cholesterol (TC) level in animals in group B compared to the control group. Animals in group D showed a decrease in total cholesterol and triglyceride levels compared to group B (untreated lead group). Groups E and F showed a significant reduction in Triglyceride level (TG) compared to lead untreated (P < 0.01). Animals in groups E and F showed an increase in the Total Cholesterol (TC) though not statistically significant and a decrease in the Triglyceride level compared to untreated lead, as shown in Table 8 below.

**Table 8.** Changes in blood lipid concentration in the experimental animals as a result of both lead toxicity and treatment.

Groups	TC (mg/dL)	TG (mg/dL)	HDL (mg/dL)	LDL (mg/dL)
**A**	2.35 ± 0.15	1.07 ± 0.15	0.75 ± 0.01	1.32 ± 0.11
**B**	2.01 ± 0.12	1.21 ± 0.11	0.58 ± 0.05	1.23 ± 0.21
**C**	1.89 ± 0.11*	1.04 ± 0.17	0.49 ± 0.04	1.24 ± 0.27
**D**	1.96 ± 0.14*	0.86 ± 0.09*	0.53 ± 0.10	1.19 ±0.13
**E**	2.42 ± 0.29**	0.78 ± 0.09*	0.54 ± 0.03	1.42 ± 0.09
**F**	2.55 ± 0.25**	0.69 ± 0.07*	0.76 ± 0.07	1.52 ± 0.11

**TC**-Total Cholesterol; **TG**-Triglyceride; **HDL**-High Density Lipoprotein and **LDL**-Low Density Lipoprotein

*Decrease compared to B at P<0.01; **Increase compared to B at P < 0.01.

### 3.8. Microscopic examination of cerebellum

The microscopic examination of the cerebellum in group A showed distinct cerebellar layers of molecular, granular, and Purkinje cell layers. The white and grey matters are consistent, while the cerebellum in group B showed severe hemorrhage vacuolation, necrosis, cell degeneration and separation of the granular layer were prominent. The result in the treated animals with piper and honey showed changes in the histoarchitecture of the cerebellum, as seen in Figures 3–5, leading to increased and improved motor activities, social behavior, memory, motivation, and social affiliation compared to group B.

**Figure 1.**
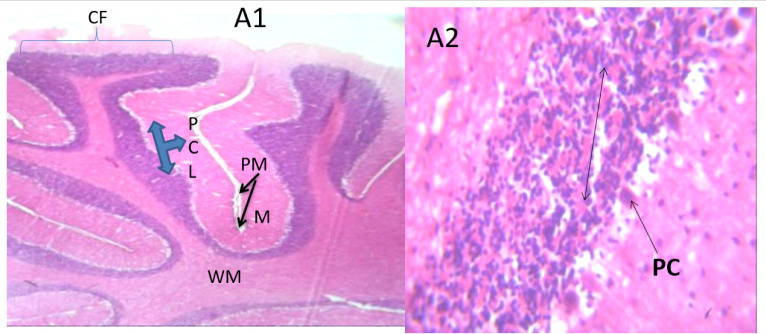
Group A cerebellum with normal cerebellar cortex (CF), purkinje cell layers (PCL), Pia matter (PM), white mater (WM), molecular layer (M), granular layer and its cells (double head arrow). X400, H & E.

**Figure 2.**
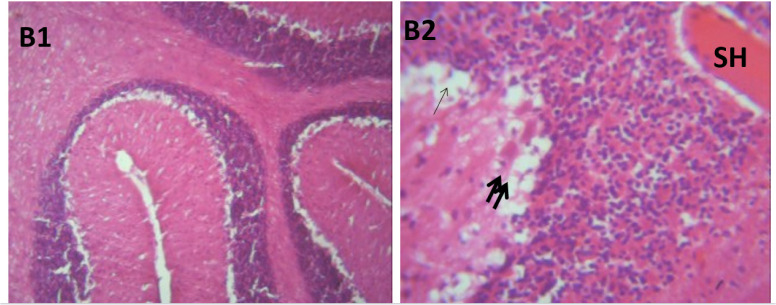
Group B cerebellum with severe hemorrhage (SH), severe vacuolation in the purkinje cell layer (thin arrow), necrotic purkinje cells (double arrows). X400, H&E.

**Figure 3.**
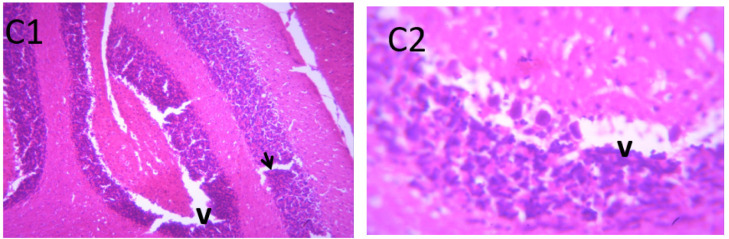
A section of group C cerebellum showing mild vacuolated (V), healthy cell and intact nuclei. (x100/400)(H/E).

**Figure 4.**
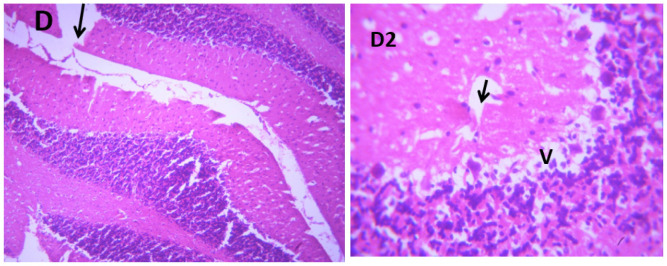
A section of group D cerebellum showing vacuolated purkinje cell layer (V), optical empty spaces due to necrosis (arrow) mild vacuolation of purkinje cell layer. (x100/400)(H/E).

**Figure 5.**
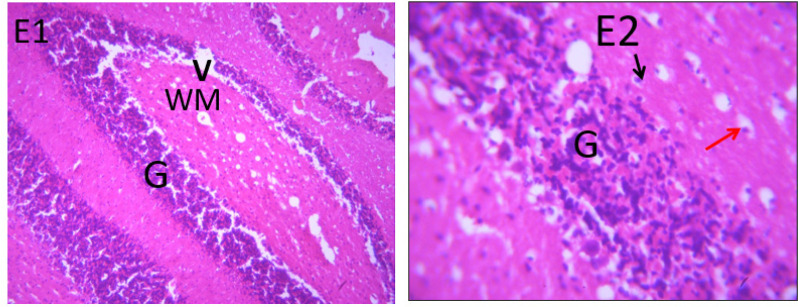
A section of group E cerebellum showing mild vacuolation of granular layer (V), white matter (WM), molecular layer cells (arrows), blood vessel (double arrows). (X100/X400)(H/E) show moderate healing with mild cystic space otherwise normal.

**Figure 6.**
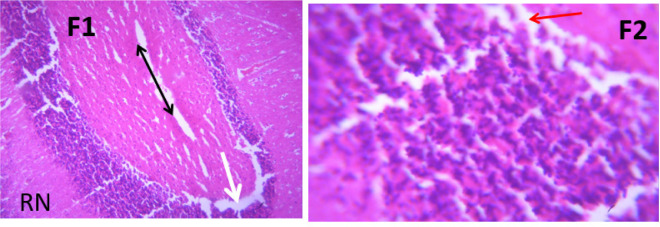
Photomicrograph of group F section of cerebellum showing mild vacuolation of Purkinje cell layer (arrows), near normal molecular cells layer (RN) and blood vessel (double head arrow); X100/X400)(H/E).

### 3.9. Microscopic examination of the hippocampus

**Figure 7.**
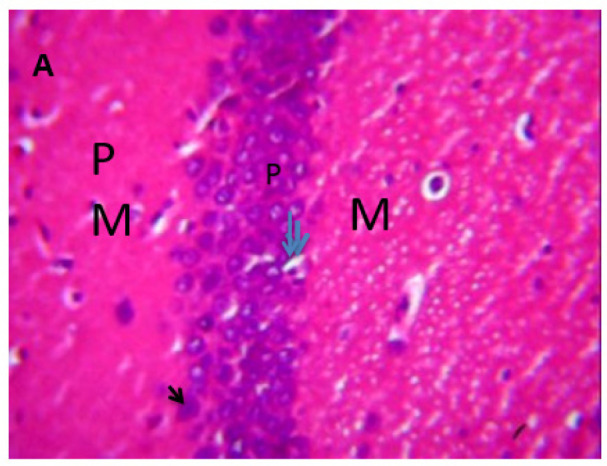
Control Group showing: polymorphic (PM), pyramidal (P), and molecular (M) layers in area CA1, triangular pyramidal cells (arrow) which is hippocampal principal cell layer with large nuclei (two arrows).

**Figure 8.**
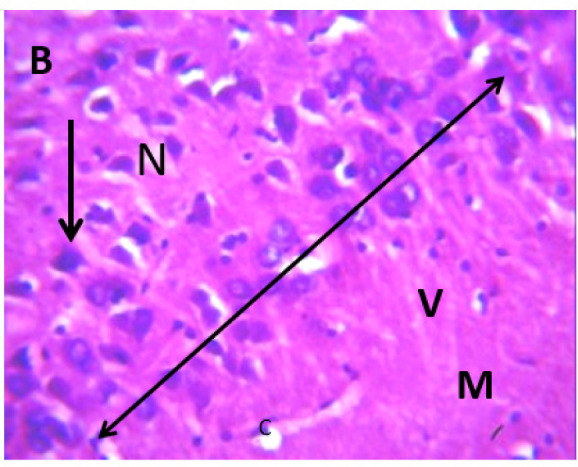
Lead untreated showing: polymorphic (pp), scattered pyramidal (double head arrow), and molecular (M) in area CA1 with few scattered cells with large nuclei (arrow) and nucleus (N), capillaries (C), shrunken pyramidal cells (V). The cell layer is sparse and not intact. H & E, 400X.

**Figure 9.**
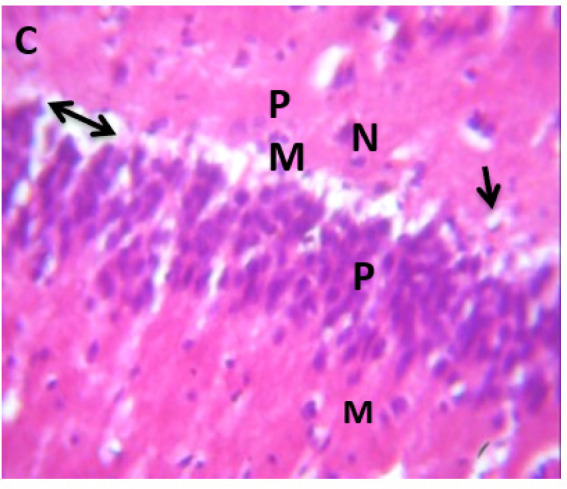
Group C, showing three layers: polymorphic (pp), pyramidal (p) and molecular (M) in area CA1, pyramidal cells with large nuclei (N), few pyramidal nerve cells with dark pyknotic nuclei and lost nuclear details (arrow), separated cell layers (double headed arrow), blood capillaries (arrow).

**Figure 10.**
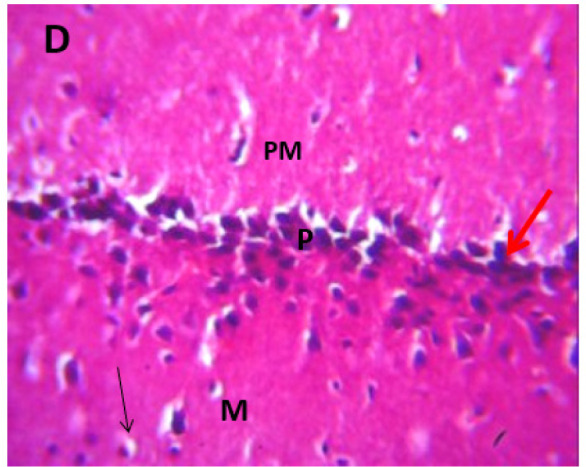
Group D showing three layers: polymorphic (pp), pyramidal (p) and molecular (M) in area CA1, pyramidal cell with large nuclei (N), few pyramidal nerve cells with dark pyknotic nuclei (double arrows), molecular cells (arrow).H & E sections and were snapped at 400X magnification.

Microscopic examination of the hippocampus showed healthy polymorphic, pyramidal, and molecular layers of the hippocampus in the control group. In contrast, lead untreated group B presented dispersed pyramidal and molecular layer, and cells with large nuclei, shrunken nerve cells, and darkly stained cytoplasm with loss of nuclear details as in Figures 7 and 8, respectively. Groups C and D presented a few pyramidal cells with dark pyknotic nuclei and loss of nuclei components with separated layers, as in Figures 9 and 10, respectively. Finally, the section from groups E and F showed mild necrotic pyramidal cells, large nuclei, and nerve cells with dark pyknotic nuclei.

**Figure 11.**
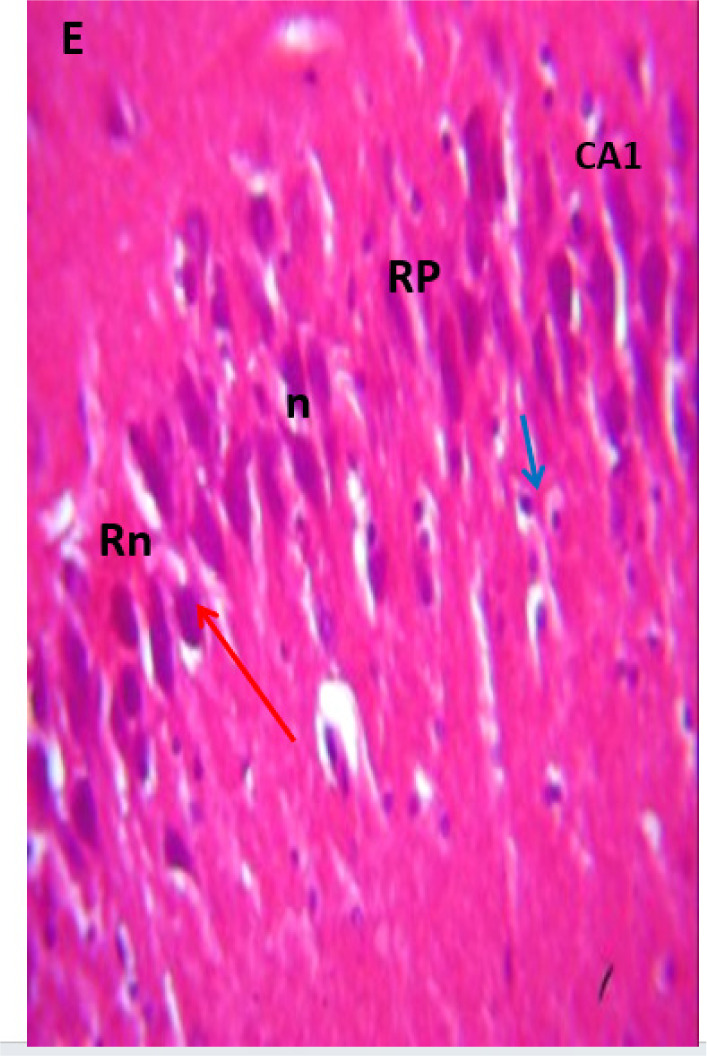
Group E showing area CA1, pyramidal cell with large nuclei (n), nerve cells with dark pyknotic nuclei (red arrow), blood vessel(blue arrow), recovering pyramidal cells (RP) and little hemorrhage (Rn).

**Figure 12.**
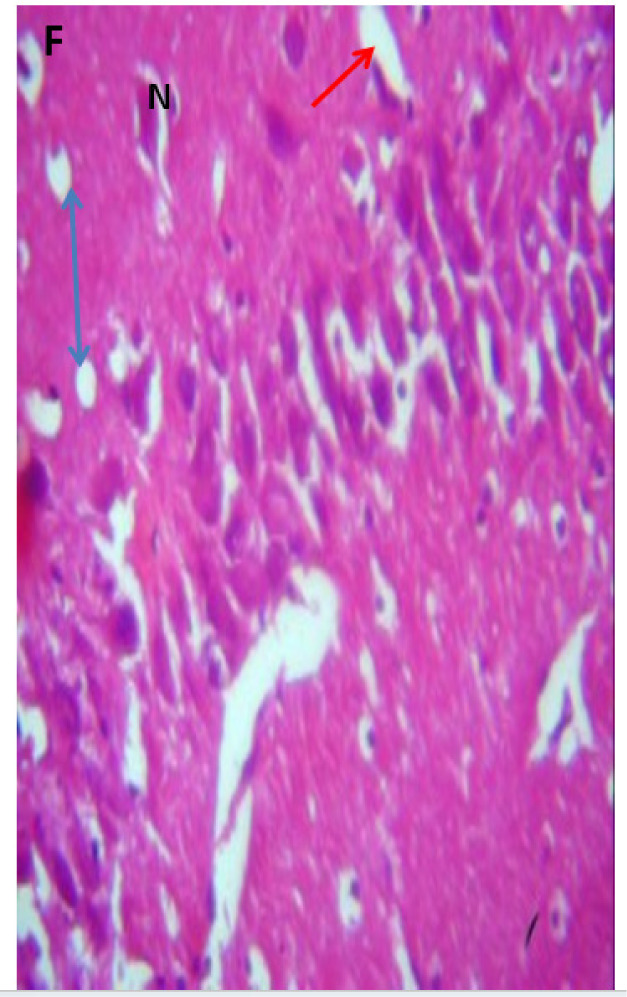
Group F showing cells with large nuclei (N), dark pyknotic nuclei (red arrow), blood vessels (double head arrow), and scattered pyramidal cells. H & E; 400X magnification.

## 4. Discussion

In this study, there was an observed loss of appetite, sluggishness, passive behavior, prolonged response to external stimuli, and a color change in feces, which was agreed upon [24]. Those findings might have led to the retarded growth and weight loss recorded in this research. Lead is known to increase the rate at which reactive oxygen species (ROS), singlet oxygen, and hydrogen peroxide are generated [25], which enhances the depletion of antioxidant defense systems, causing damage, according to Lucke-Wold et al. [26]. In agreement with Sharma et al. [27], the decrease in GPx and GOT levels in this research is a pointer to the depleting action of lead. Upon the administration of honey, GPx significantly increased, corroborating Samarghandian et al. [28].

In contrast, combined administration increased both GPx and GOT, as a pointer that they can enhance the immune system depleted by lead and rebuild the antioxidant system (table 2). The high honey's effectiveness helped raise GOT and GPx when singly administered to the rats, as seen in table 2 above. In agreement with the report of Amadi et al. [29] and Blake et al. [30], the reduction in cholesterol and triglyceride levels in medium and high doses. *Piper guineense* is its antioxidant potential capable of preventing lipid peroxidation, thereby reducing cholesterol levels.

As seen in Tables 5 and 6, the increase in mobility of untreated rats may indicate increased locomotive activity as a sign of survival strategy developed by the animals in combating the depression induced by lead intoxication [31],[32]. In the treated rats, mobility increased significantly after receiving P. guineense and honey, suggesting a loss of hope due to depression caused by lead intoxication in experimental animals. The reduced grip loss latency indicates compromised muscle strength and the ability to grasp and hold onto objects. At the same time, decreasing scores on limb impairment indicate compromised limb function and resilience showed by rats induced with lead in this research, as seen in tables 3 and 4, agreeing with Weilch et al. [33]. This finding suggests that increased latency to grip might be due to the therapeutic effect of *P. guineense* and honey on muscle coordination.

In contrast, the reduced latency in the lead untreated might indicate compromised limb functionality and strength [34]. It has been noted that social recognition plays a critical role in the structure and stability of the networks and relationships that define societies [35]. Various neuropsychiatric disorders are characterized by social behavior and recognition alterations, including depression, autism spectrum disorders, bipolar disorders, obsessive-compulsive disorders, and schizophrenia [36]. The control group spent more time in isolation and minimal time with the unfamiliar rat and omit more time with the familiar rat, indicating standard social memory, motivation, and affiliation. The lead-untreated group spent little time with the familiar rat while spending a lot of time in isolation as a sign of impairment or decreased social motivation and affiliation response which might be due to toxicity. The results in Tables 2 and 3 suggest that honey and P. guineense are ineffective in restoring social motivation, affiliation, and memory in their separate states [20]. The propensity to be comfortable and spend time with strangers rather than familiar ones is referred to as social novelty [36]. The control rats preferred familiar rats to novel rats, indicating social memory with no preference for novelty (Tables 2 and 3). In contrast, the untreated rats' ability to spend more time with novel rats shows their affinity for novel experiences with poor social motivation, memory, and affiliation, in agreement with Moy et al. (34). The rats were able to spend more time with familiar rats after being treated with the extract and honey, which indicates a slow predilection for novel experiences and spatial learning.

Microscopically, there were various layers of the hippocampus and CA1 to CA4 in control, while lead caused varied alterations to the tissues, as seen in Figures 6 and 7 [37]. The untreated lead group displayed neuronal damage to the hippocampus characterized by a scattered pyramidal layer which contains the principal cells and molecular layer, cells with large nuclei, numerous shrunken pyramidal nerve cells with darkly stained cytoplasm and lost nuclear details, and a sparse cell layer. The *p. guineense* as an antioxidant helped restore the alterations caused by lead toxicity in the hippocampus, as seen in Figures 8 and 9. These restorations inform of healing recorded in the treated sections is a pointer towards the anti-inflammatory role of the extract, which may have helped improve the animal's memory, and social behavior, and the reduction is the lipid peroxidation as seen in Tables 1, 2, and 8. The effect of piper and honey on the hippocampal sections is an excellent pointer towards their potential in restoring neuronal degenerations caused by heavy metal toxicity. The microscopic sections of the cerebellum are represented in Figures 1–6 above, with the control showing normal histoarchitecture (Figure 1A) and several alterations in lead untreated (Figure 2B). The severe hemorrhage seen in Figure 2 could be due to blood vessel rupture and neuronal damage caused by lead intoxication which also affected their motor activities such as limb impairment, grip strength, and mobility, as seen in Tables 3, 4, and 5 above [38]. The control presented a section of the cerebellum that looked very healthy, with all the cells in the layers intact [39]. The rats that received the extract and honey showed unprecedented healing and cell regeneration across all the layers, as evidence of the anti-inflammatory and healing properties of *P. guineense* and honey. The restoration seen on the sections may have been instrumental in the improved muscle tone translating to improved grip strength, limb functionality, and mobility of the animals (Tables 4 and 6). This improvement may have been due to the reduced lipid peroxidation due to decreased production of ROS and singlet oxygen in the brain's cells by anti-inflammatory substances, as reported by Lucke-Wold et al. [40].

## 5. Conclusion

The neurotoxic effects of lead are evidenced by impaired social behavior, reduced motor activity, decreased GPx and GOT concentration, distorted hippocampal and cerebellar histoarchitecture, and lipid peroxidation. However, in a dose-dependent manner, the antioxidative and anti-inflammatory properties of *P. guineense* and honey in a combined state proved very effective in alleviating the alteration caused by lead toxicity.
